# A Curious Case of Crystal Deposit Disease in the Petrous Bone

**DOI:** 10.7759/cureus.6375

**Published:** 2019-12-13

**Authors:** Marije De Jong, Carlos Candanedo, Tal Keidar Haran, Michal Kaufman

**Affiliations:** 1 Otolaryngology Head and Neck Surgery, Hadassahe Hebrew University Medical Center, Jerusalem, ISR; 2 Neurosurgery, Hadassah-Hebrew University Medical Center, Jerusalem, ISR; 3 Pathology, Hadassah Hebrew University Medical Center, Jerusalem, ISR; 4 Otolaryngology Head and Neck Surgery, Hadassah Hebrew University Medical Center, Jerusalem, ISR

**Keywords:** calcium deposition disease, petrous bone, tumoral calcinosis, pseudogout

## Abstract

Crystal deposit disease is a rare disorder with benign dense soft tissue calcium containing accumulations presenting as pseudogout or tumoral calcinosis. It rarely affects the head and neck region and even less to the petrous bone. We describe a case of para-articular tumoral calcinosis involving the external auditory canal wall in close proximity to the temporomandibular joint with extension towards the middle cranial fossa floor in a 73-year-old man presenting with otalgia and progressing mixed hearing loss. Subtotal petrosectomy with obliteration of the middle ear and mastoid was done with complete removal of the lesion. We discuss the course, treatment and final pathology with possible explanations for the pathophysiology in this particular case. Although tumoral calcinosis is uncommon, this entity should be considered in the differential diagnosis when an osteogenic temporal lesion is seen on computed tomography or magnetic resonance imaging. The treatment for this benign tumor includes complete excision of the lesion in symptomatic cases. Proper evaluation including anamnesis of the family history and previous trauma as well as serology should be done. The exact etiology and classification of crystal deposit diseases require further study.

## Introduction

Osteogenic and chondrogenic lesions involving the temporal bone are infrequent and include fibrous dysplasia, osteoma, ossifying fibroma and some other less common pathologies. A rare pathology is crystal deposit disease. Benign dense soft tissue calcium containing deposits are referred to as tumoral calcinosis and are build up from calcium hydroxyapatite crystals [1]. It often affects joints such as the hips, elbows and shoulders, but is rarely found in other sides [2,3]. In some cases, the temporal mandibular joint is involved as pseudogout with the accumulation of calcium pyrophosphate dihydrate (CPPD) crystals [1]. We present a rare case of para-articular tumoral calcinosis of the petrous bone, involving the middle cranial fossa floor in a 73-year-old man. We discuss the course, treatment and final pathology with possible explanations for the pathophysiology in this particular case. 

## Case presentation

**Figure 1:**
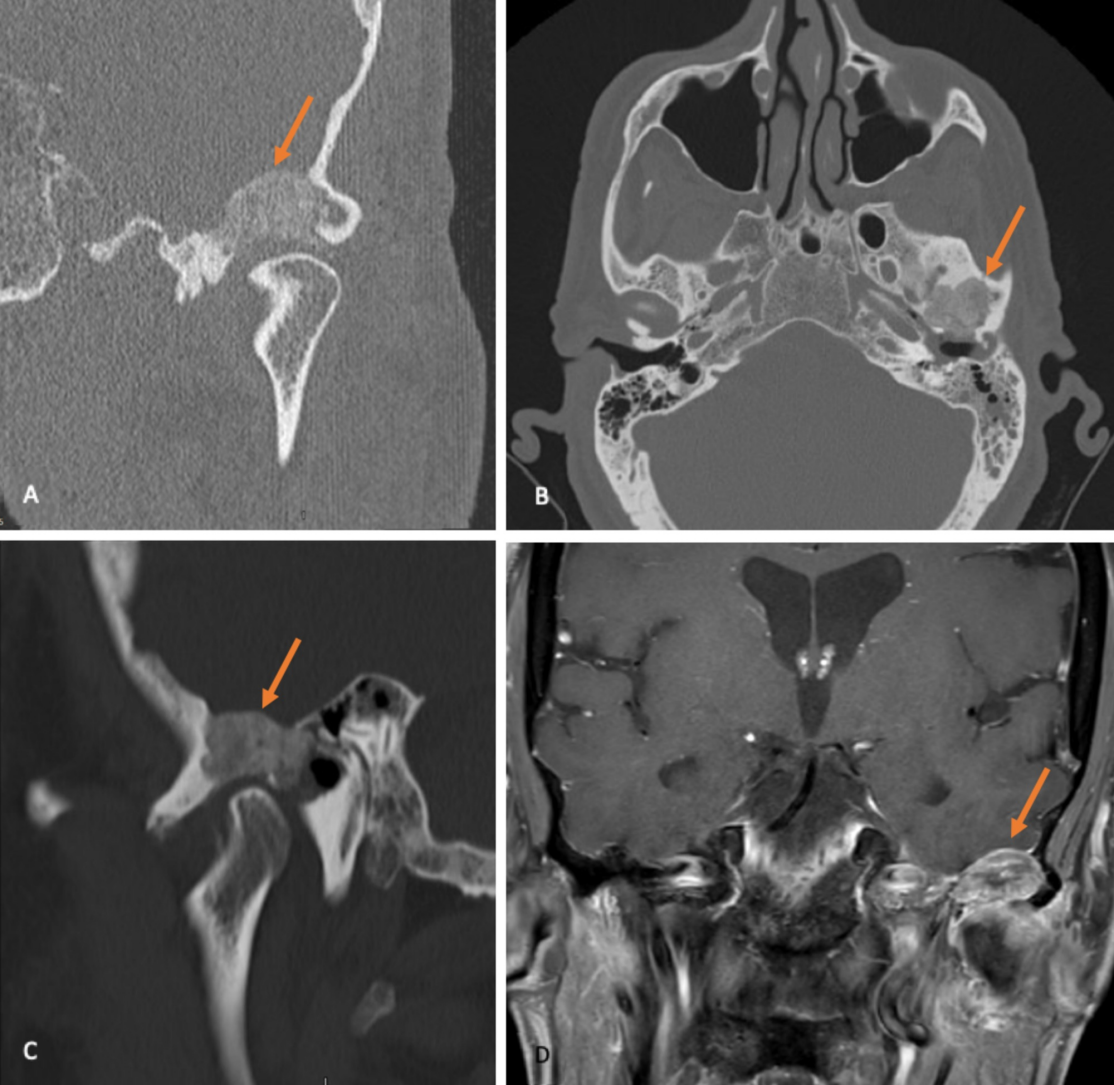
Preoperative imaging left temporal area. Non-contrast head CT: (A) coronal view of a left para-articular temporal lesion and (B) axial view showing erosion of the anterior external auditory canal wall and opacification of the mastoid air cells; (C) sagittal view and (D) sagittal T1-weighted MRI with gadolinium showing lesion extension towards the middle cranial fossa apparently without signs of dural or cerebral involvement.

A 73-year-old man initially presented to the emergency department with complaints of twitching of the mouth and the sensation of fullness in the left ear. After a neurologist examination a head computed tomography (CT) angiogram was performed revealing opacification of the left mastoid cells with a bony lesion at the anterior aspect of the external auditory canal (Figure 1A).

**Figure 2:**
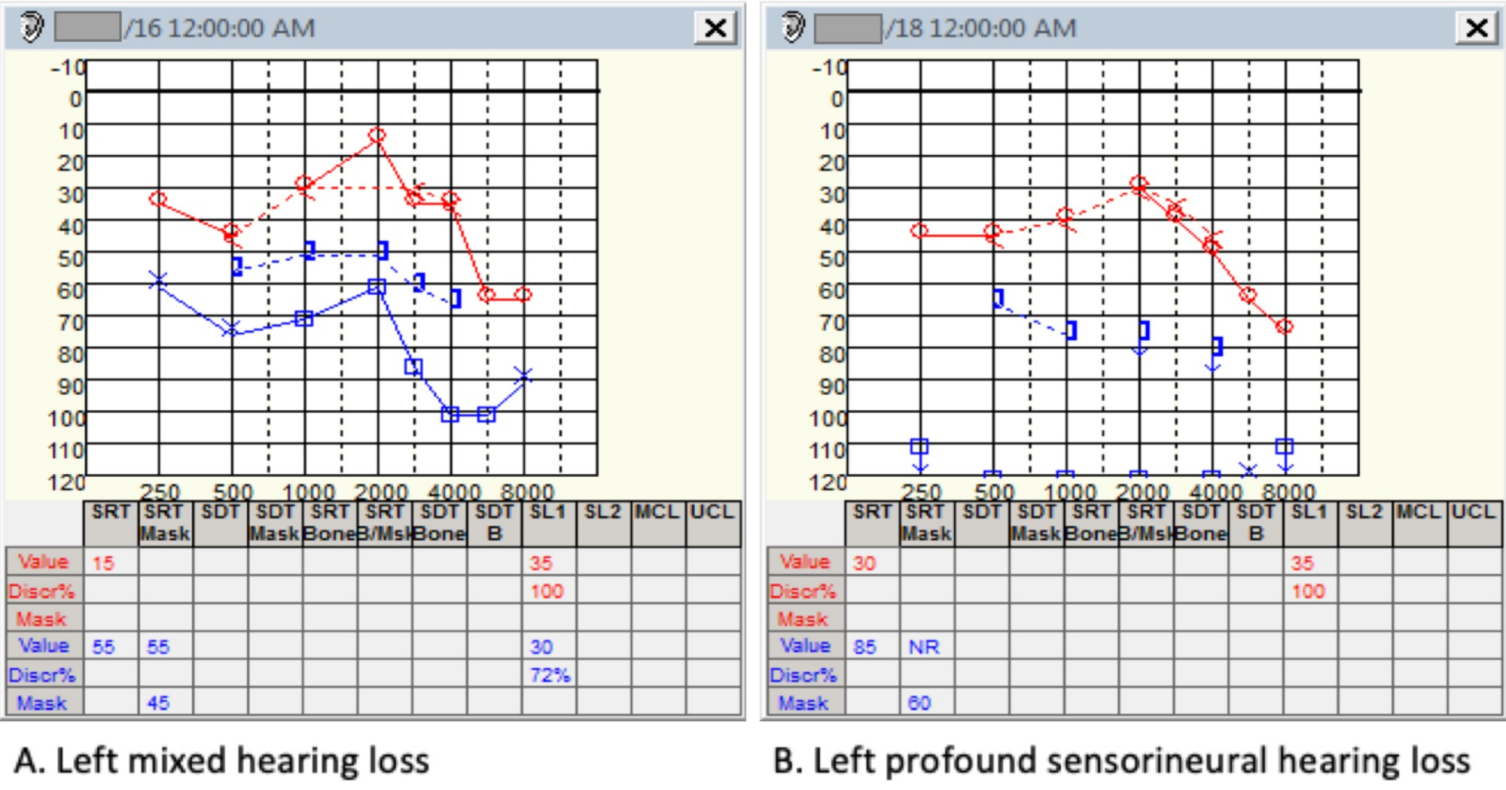
Preoperative audiometry with gradual perceptive worsening.

The lesion was suspicious for fibrous dysplasia. One month after the start of his symptoms, he was admitted to the otolaryngology ward for the evaluation of long-standing dizziness and imbalance. Physical examination showed a clinical presentation of serous otitis media for which a myringotomy was done. Tuning fork tests and an audiogram confirmed a left mixed hearing loss (Figure 2A). A fistula test on the left showed a right beating nystagmus, and caloric testing was normal. Posturography was indicative of both peripheral and central involvement. There was no other cranial nerve deficit. Oral prednisone 60 mg once daily was started for a week and betahistine for symptomatic relief. Repeated audiometry showed a gradual worsening of asymmetric left sensorineural hearing loss (Figure 2B).

Seven years later the patient was re-evaluated with a head CT and brain magnetic resonance imaging (MRI) for new onset of left otalgia and pain radiating to the temporomandibular joint. The bony lesion at the anterosuperior aspect of the external auditory canal increased in size to 18 mm and extended into the middle cranial fossa floor, with no dural invasion (Figure 1B-1D).

 

**Figure 3:**
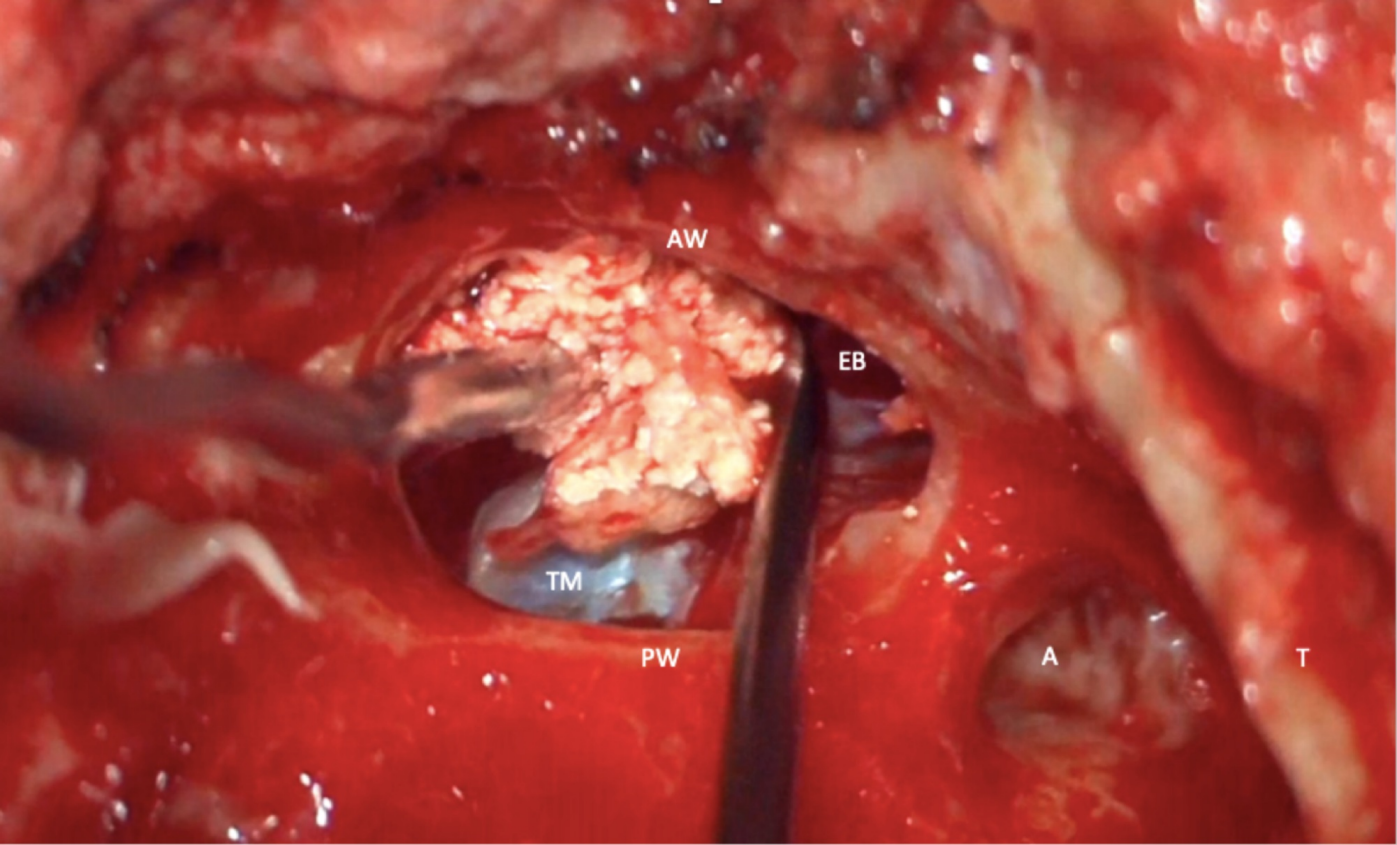
Intraoperative image of whitish debris coming from an eroded cavity anterior from the left external auditory canal (EAC). AW = anterior wall of EAC; PW = posterior wall of EAC; TM = tympanic membrane; A = antrum; T = tegmen mastoideum; EB = eroded bone of EAC wall

During otoscopic examination it was noted that the external auditory canal had become obstructed by anterior bulging. A biopsy was taken by the transmeatal approach. The pathology result of the soft white tissue came back as cholesteatoma. For this reason, a non-echo planar diffusion-weighted MRI for cholesteatoma detection was done; however, no restriction specific for cholesteatoma was noted. Based on the clinical complaints and radiologic enlargement of the tumor, an excisional biopsy was recommended. Under general anesthesia with facial nerve monitoring, the patient underwent a subtotal petrosectomy with resection of the lesion and obliteration of the middle ear and mastoid with blind sac closure of the external auditory canal. A far retroauricular skin incision was made extending towards the more anterior temporal area. Blind sac closure of the external auditory canal was performed, a radical mastoid cavity was created, and the mastoid and middle ear showed granulation tissue. During this surgical step, bone erosion was noted at the anterior external auditory canal wall in close proximity to the tympanic membrane. After widening the erosion, white crumbling debris was removed from a preauricular area that extended towards the middle cranial fossa and temporomandibular joint (Figure 3); however, the latest one was not involved by the lesion and the middle cranial fossa dura was intact.

**Figure 4:**
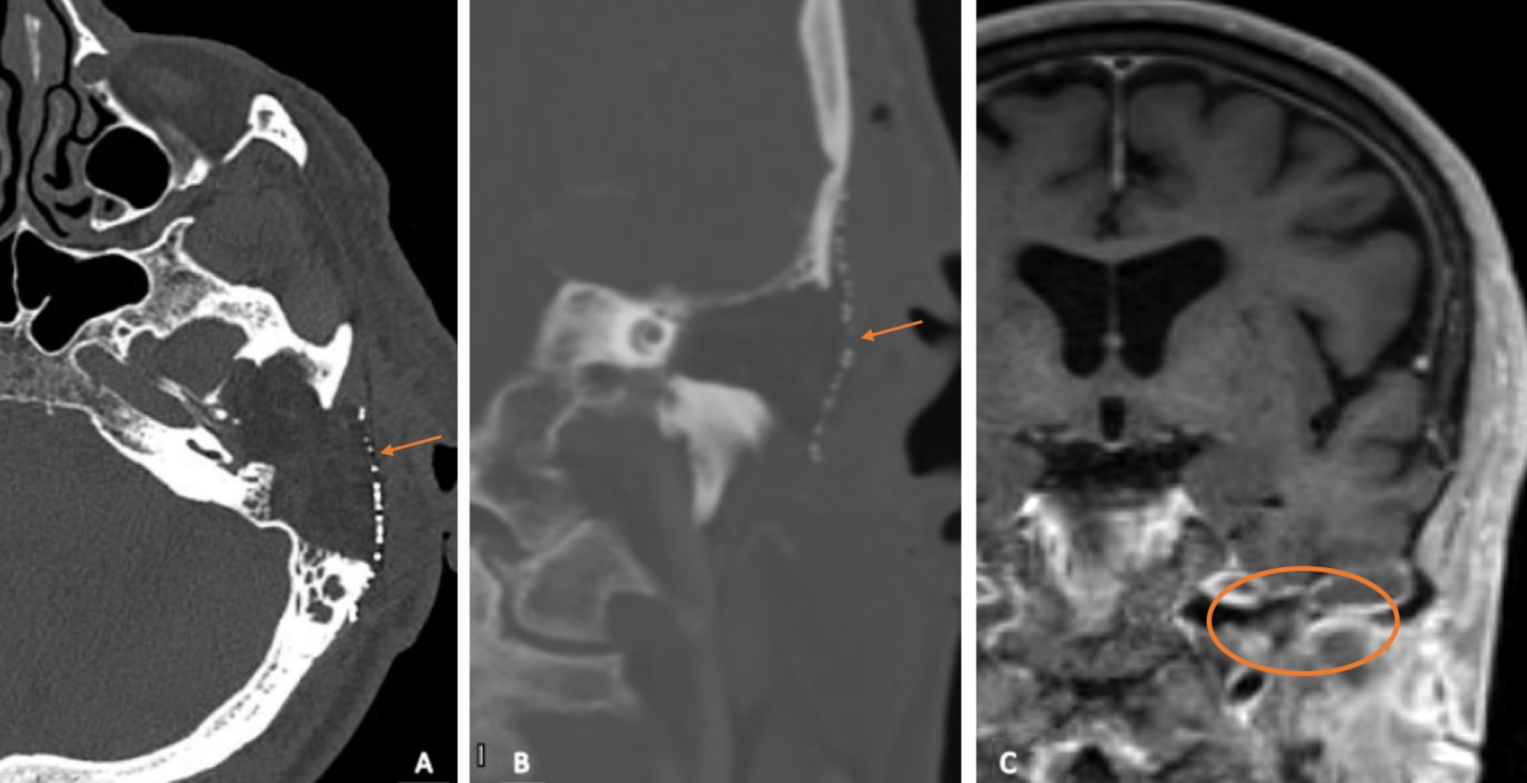
Postoperative imaging. High-resolution CT ears with contrast: (A) axial and (B) coronal view showing the subtotal petrosectomy cavity obliterated with abdominal fat and covered by a mesh plate (arrows). (C) Coronal T1-weighted gadolinium-enhanced MRI showing complete removal of the tumor (circle).

Intraoperative frozen section pathology report was inconclusive but indicated an inflammatory process. After complete removal, the remaining petrous cavity was filled with previously harvested abdominal fat and a mesh plate was used to keep the graft in place. The wound was closed in layers without any sign of cerebrospinal fluid (CSF) leak during the procedure. Postoperative CT and MRI showed a stable obliterated cavity without signs of residual tumor (Figure 4). 

**Figure 5:**
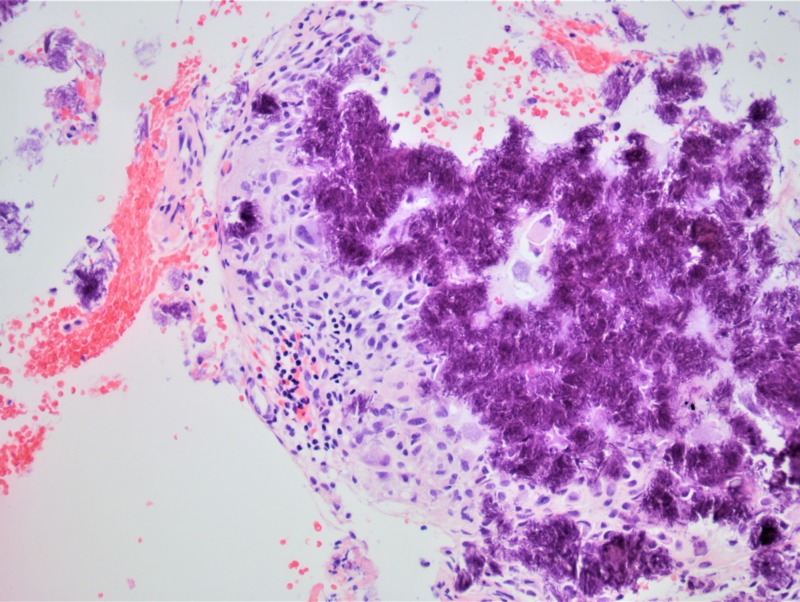
Pathology slide. Extensive calcium crystal deposition with giant cell reaction. H&E stain. X20.

**Figure 6:**
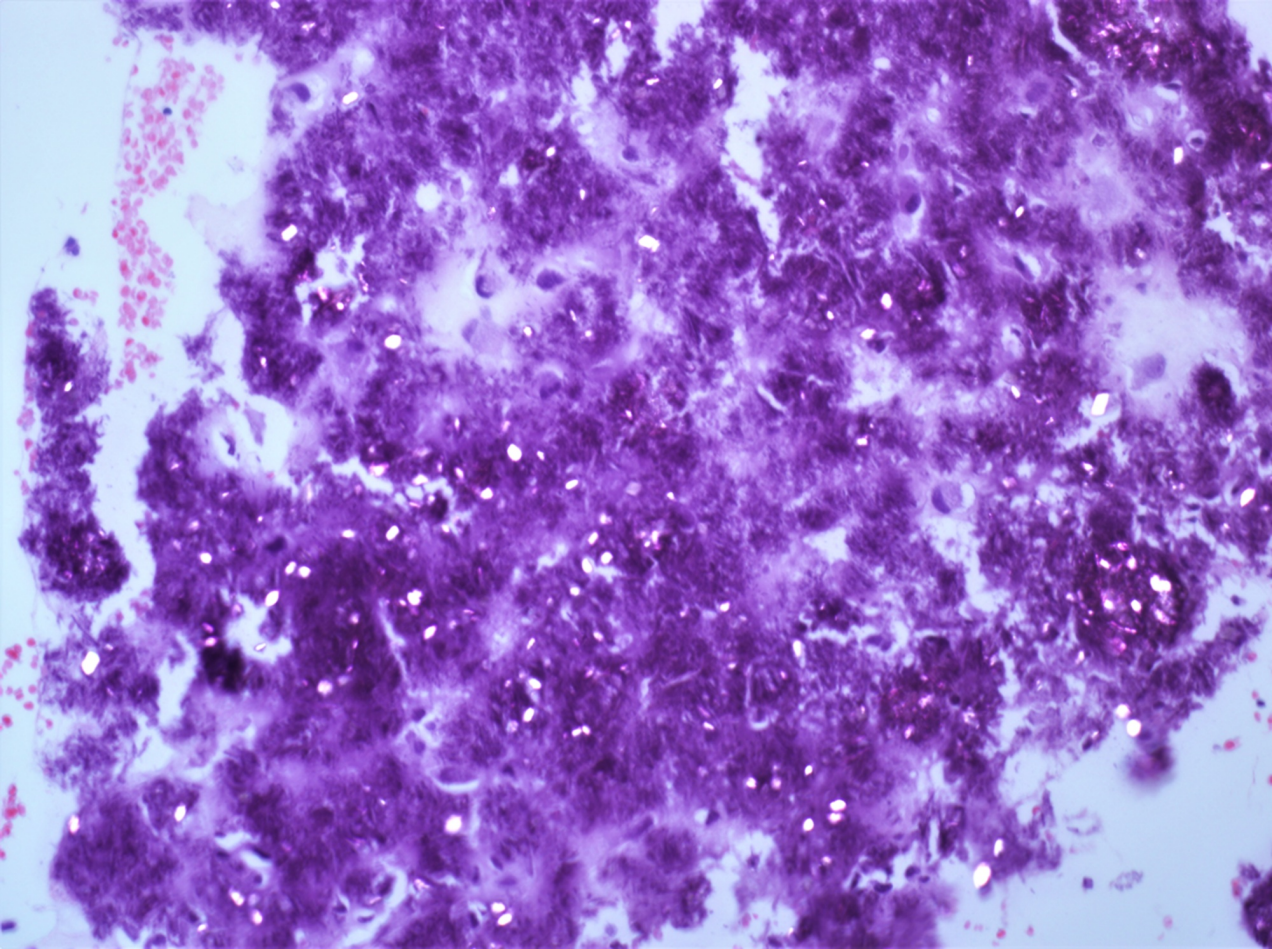
Pathology slide. Weakly birefringent rhomboid calcium pyrophosphate dihydrate crystals under polarized microscopic examination. H&E stain. X20.

The postoperative course was uneventful with mild dizziness for two days but no further imbalance. The final histopathology examination mentioned fragments of cartilaginous and periosteal tissue with extensive calcium crystal deposition seen on polarized light (Figure 5), compatible with calcium pyrophosphate deposition (Figure 6).

## Discussion

The classification of crystal deposit disease is somewhat confusing. Pseudogout refers to CPPD crystal deposits, whereas tumoral calcinosis is the accumulation of calcium hydroxyapatite crystals. However, CPPD crystal deposits in tumoral calcinosis has been described [1]. In the present case, the tumor was located in close proximity to the temporomandibular joint but did not involve intra-articular cartilage and synovia as seen in pseudogout. One case of CPPD in the middle ear has been mentioned in the literature; however, this originated from the incudomalleolar joint as pseudogout [4]. Therefore, classifying the lesion in our case as tumoral calcinosis in the periarticular tissues with CPPD crystals seems appropriate. 

While most cases of tumoral calcinosis are sporadic, an autosomal recessive inheritance pattern has been described [1]. Tumoral calcinosis likely results as a secondary phenomenon to local tissue injury, connective tissue disease or metabolic disturbances such as hyperparathyroidism or chronic renal failure resulting in hyperphosphatemia [1,2,4]. It is therefore important to ask about history of trauma and family members with joint disease. Serum calcium levels, serum phosphorus levels and renal function tests should be carried out. In our particular case, serum calcium and phosphate levels were normal, with normal renal function and no family history of joint disease. Our patient could not recall any head trauma that can fit the hypothesis of a trauma-related pathophysiology. In our conclusion, it presented as a true idiopathic tumoral calcinosis. 

Another finding that raises questions is the progressive mixed hearing loss. Although the conductive component can be partially explained by occlusion of the external auditory canal and the non-aerated middle ear and mastoid, a progressive sensorineural decline is unlikely related to the gradual enlargement of the tumor or local mass effect. A shared longstanding inflammatory process for both pathologies can be postulated; however, there is a lack of evidence for such hypothesis. At the time of the surgery, there was no functional hearing in the left ear. 

Tumoral calcinosis can grow fast with inflammation or with a more chronic course over a period of several months [5]. Although the lesions are usually painless, they can become symptomatic because of compression and disruption of surrounding tissues. Surgical excision should be considered treatment of choice in symptomatic cases. Incomplete excision can lead to recurrences [3]. We chose a subtotal petrosectomy with obliteration and blind sac closure since hearing preservation was not of consideration and it might reduce the risk of future paradoxical CSF leak and meningitis because of the lesion’s involvement into the middle cranial fossa.

## Conclusions

We present a case of a calcium containing lesion of the petrous bone in close proximity to the temporomandibular joint with extension towards the middle cranial fossa floor. Although rare, this entity should be considered in the differential diagnosis when an osteogenic temporal lesion is seen on CT or MRI. Proper evaluation including anamnesis of the family history and previous trauma as well as serology should be done. The exact etiology and classification of crystal deposit diseases require further study. The treatment for this benign tumor includes radical excision of the lesion itself in symptomatic cases.
